# Supplementary material for: Elements and evolutionary determinants of genomic divergence between paired primary and metastatic tumors
Source: PLoS Comput Biol. 2021 Mar 17;17(3):e1008838. doi: 10.1371/journal.pcbi.1008838 (PMC8007046; doi:10.1371/journal.pcbi.1008838)
Supplement: S1 Appendix — (PDF) [file pcbi.1008838.s001.pdf]

Elements and Evolutionary Determinants of Genomic Divergence Between Paired Primary and Metastatic Tumors

S1 Appendix

Ruping Sun[1,2◕*], Athanasios N. Nikolakopoulos[1,2◕]

1 Department of Laboratory Medicine and Pathology, University of Minnesota, Minneapolis, MN, USA
2 Masonic Cancer Center, University of Minnesota, Minneapolis, MN, USA

◕These authors contributed equally to this work.
* ruping@umn.edu

Derivation of Formula (6) of the main document

We define the events

$$U_i = \{\text{variant } c_i \text{ is not detectable}\}$$
$$D_i = \{\text{variant } c_i \text{ is detectable}\}$$

For $j = 0, \ldots, k_{\text{seed}} - 1$ we have

$$\begin{aligned}
\Pr[B_{md}^{k_{\text{seed}}} = k_{\text{seed}} - j] &= \Pr(D_j \cap U_{j+1} \cap \ldots \cap U_{k_{\text{seed}}}) \\
&= \Pr(D_j | U_{j+1} \cap \ldots \cap U_{k_{\text{seed}}}) \prod_{k=j+1}^{k_{\text{seed}}} \Pr\left(U_k \middle| \bigcap_{i=k+1}^{k_{\text{seed}}} U_i \right) \qquad (1) \\
&= \Pr(D_j \cap U_{j+1}) \qquad \text{(infinite allele model assumption)} \\
&= \Pr(U_{j+1}|D_j) \Pr(D_j) \\
&= \Pr(U_{j+1}|D_j) \Pr(D_j) + \Pr(U_j) - \Pr(U_j) \\
&= \Pr(U_{j+1}|D_j) \Pr(D_j) + \Pr(U_j) \Pr(U_{j+1}|U_j) - \Pr(U_j) \\
&= \Pr(U_{j+1}) - \Pr(U_j) \qquad (2) \\
&= d_{c_j} - d_{c_{j+1}} \qquad (3)
\end{aligned}$$

For $j = k_{\text{seed}}$, we define $\Pr[B_{md}^{k_{\text{seed}}} = k_{\text{seed}} - j]$ as

$$\Pr[B_{md}^{k_{\text{seed}}} = 0] = \Pr(D_j) = d_{c_j} \qquad (4)$$

The following lemma proves that the probability mass functions constructed as above are well-defined for all values $k_{\text{seed}} \in \mathbb{N}_+$.

Lemma 1. *Assuming* $\mathrm{P}(D_0) = 1$, *then for all* $k \in \mathbb{N}_+$ *it holds*

$$\mathrm{P}(D_k) + \sum_{j=0}^{k-1} \mathrm{P}(D_j \cap U_{j+1}) = 1 \qquad (5)$$

Proof. For $K = 0$ it holds trivially since $\mathrm{P}(D_0) = 1$. For $K = 1$ we have

$$\begin{aligned} \mathrm{P}(D_1) + \sum_{j=0}^{0} \mathrm{P}(D_j \cap U_{j+1}) &= \mathrm{P}(D_1) + \mathrm{P}(U_1) - \mathrm{P}(U_0) \\ &= \mathrm{P}(D_1) + \mathrm{P}(U_1) = 1 \end{aligned} \tag{6}$$

Let us assume that it holds for $K = k$. We will show that it holds for $K = k + 1$.

$$\begin{aligned} \mathrm{P}(D_{k+1}) + \sum_{j=0}^{k} \mathrm{P}(D_j \cap U_{j+1}) &= \mathrm{P}(D_{k+1}) + \mathrm{P}(D_k \cap U_{k+1}) + \underbrace{\sum_{j=0}^{k-1} \mathrm{P}(D_j \cap U_{j+1})}_{1-\mathrm{P}(D_k)} \\ &= \mathrm{P}(D_{k+1}) + \mathrm{P}(U_{k+1}) - \mathrm{P}(U_k) + 1 - \mathrm{P}(D_k) \\ &= 1 \end{aligned} \tag{7}$$

And the proof is complete.

□
