# Supplementary figures and images for: Elements and evolutionary determinants of genomic divergence between paired primary and metastatic tumors
Source: PLoS Comput Biol. 2021 Mar 17;17(3):e1008838. doi: 10.1371/journal.pcbi.1008838 (PMC8007046; doi:10.1371/journal.pcbi.1008838)

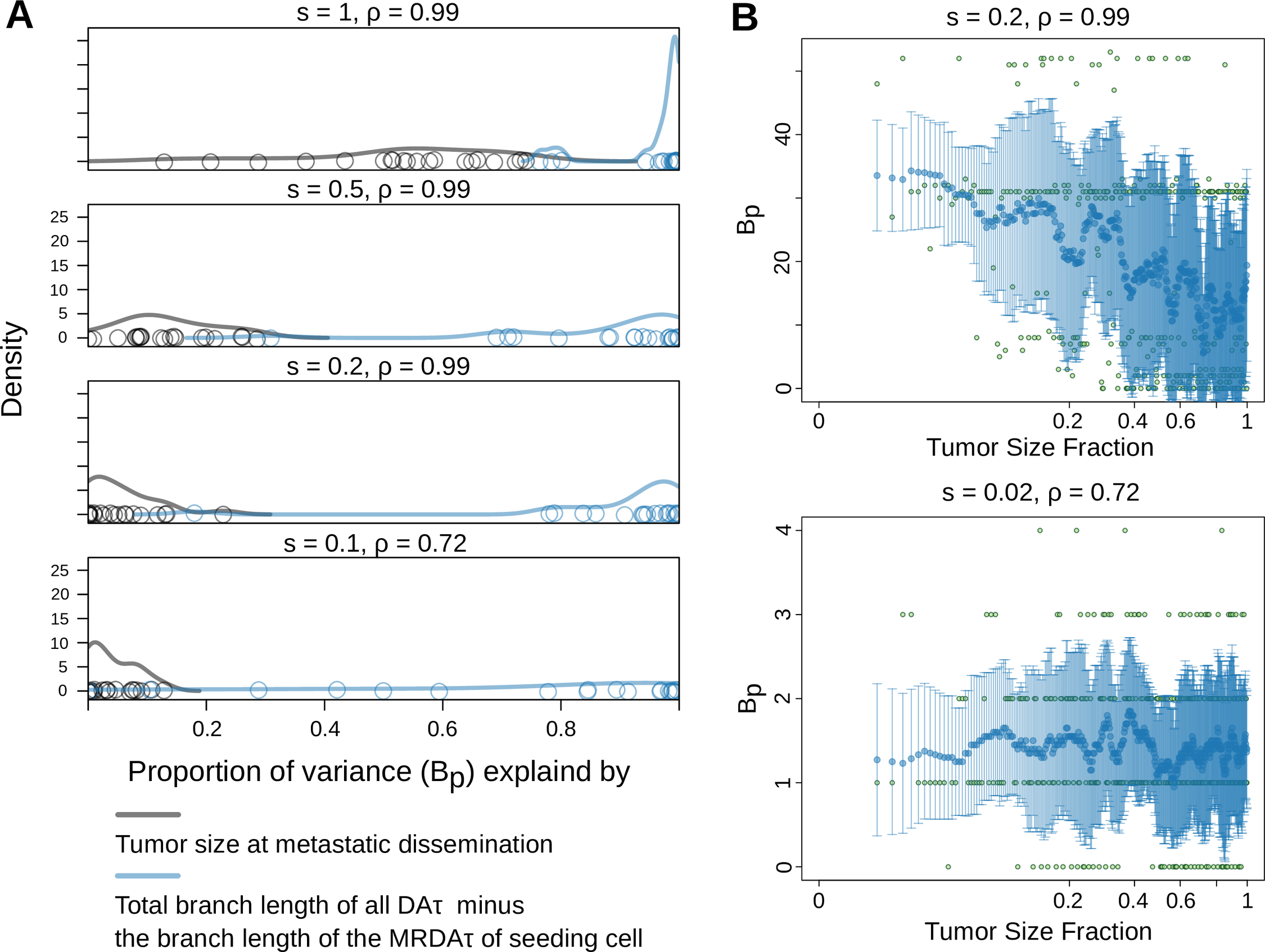

Supplement: S1 Fig — (A) The single-cell evolutionary tree based definition of Bp: the total branch length of all Detectable Ancestors at γ (DAγ) minus the branch length of the most recent detectable ancestor (MRDAγ) of the metastatic seeding cell, captures the majority of the variance of Bp measured from the virtual tumors (blue density curves). By contrast, the tumor size at metastatic dissemination, as a surrogate of dissemination time (gray density curves) has much less explanatory value for Bp. Note that we focus on moderate and strong selection only, since the variability of Bp under weak selection (s < 0.05) is negligible. (B) Two virtual tumors with distinct kinetics exemplify the conditional dependence of Bp on metastatic dissemination time. The measured Bp from virtual tumors is plotted against the tumor size fraction when the seeding cell (green dots) disseminates, the running mean (blue dots) and standard deviation (blue bars) of Bp is also shown. (TIF) [file pcbi.1008838.s002.tif]

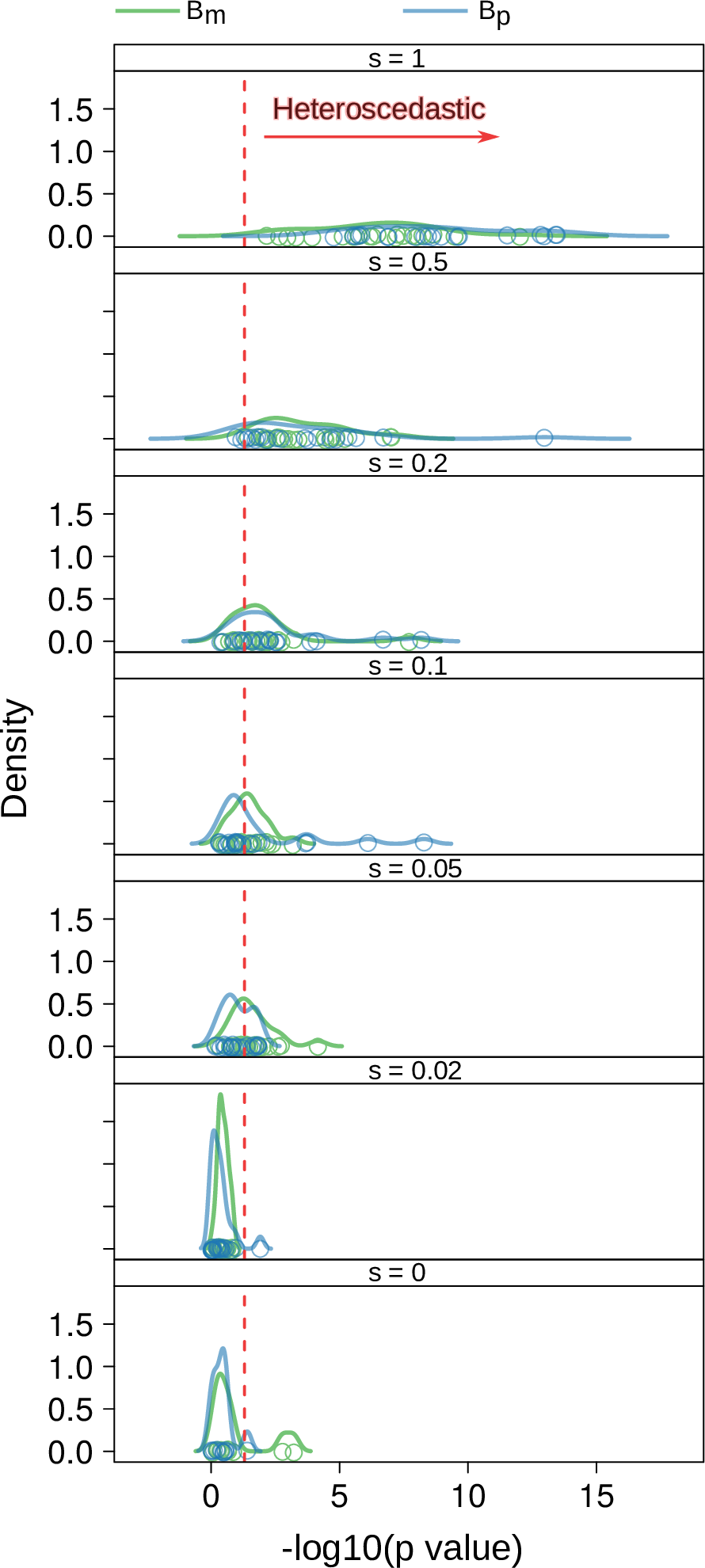

Supplement: S2 Fig — The time-ordered vector of Bm and Bp are heteroscedastic under growth modes involving strong selection (s ≥ 0.1). The p-value of White’s test for heteroscedasticity [41] is shown at negative log10 scale. A p-value of 0.05 is marked as the dashed red vertical line. (TIF) [file pcbi.1008838.s003.tif]

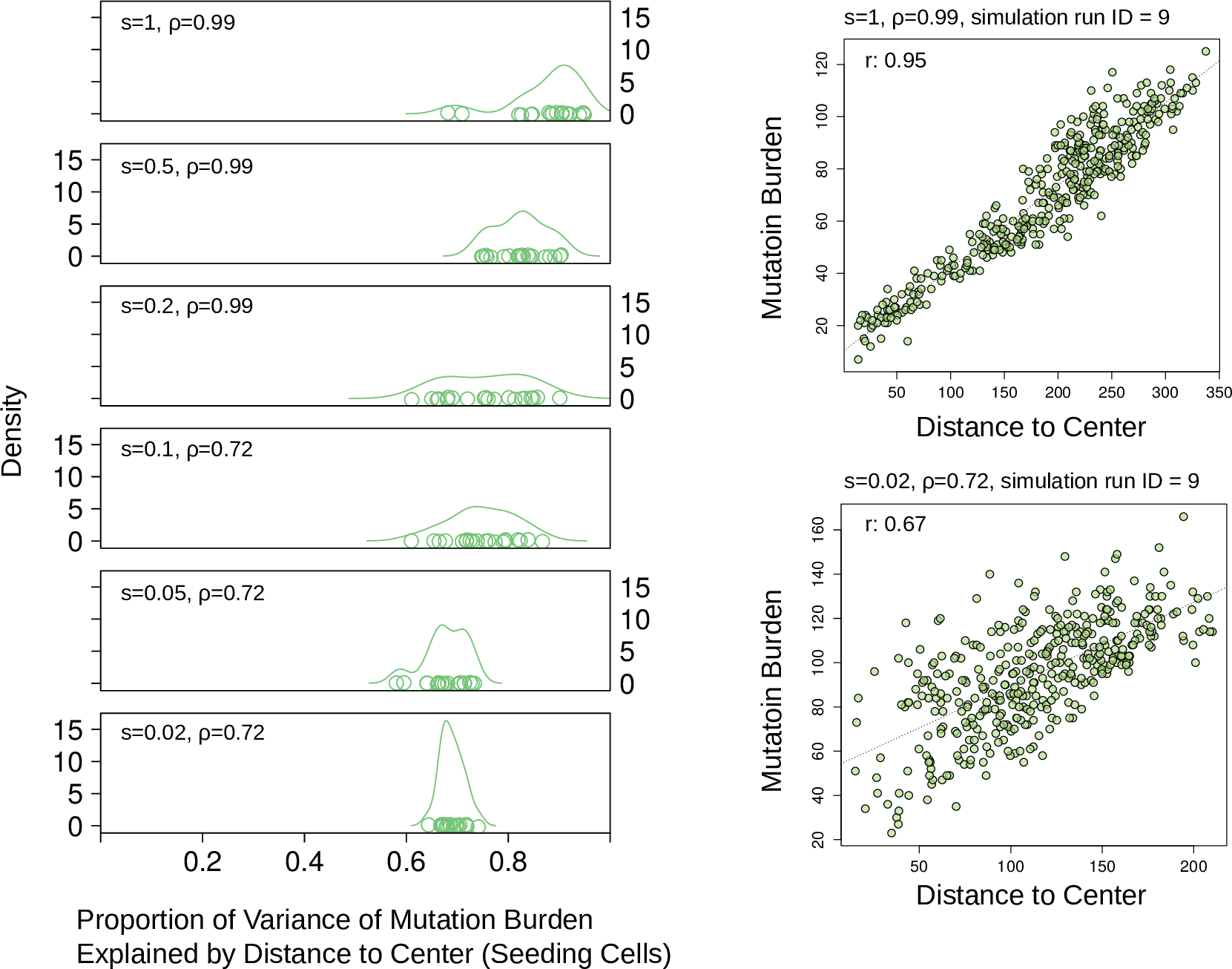

Supplement: S3 Fig — We used the Euclidean distance of a cell to the center of the simulation lattice (Distance to Center) to plot the spatial cell genealogy trees (Figs 3B and 5B in the main manuscript). Here it is shown that the majority of the variance of this distance metric is captured by the mutation burden across our simulations (left panel). The right panel shows such strong positive correlations for two example simulation runs with distinct selection coefficients. This pattern is consistent with peripheral growth, attributable to the spatial constraints in the computational modeling. (TIF) [file pcbi.1008838.s004.tif]

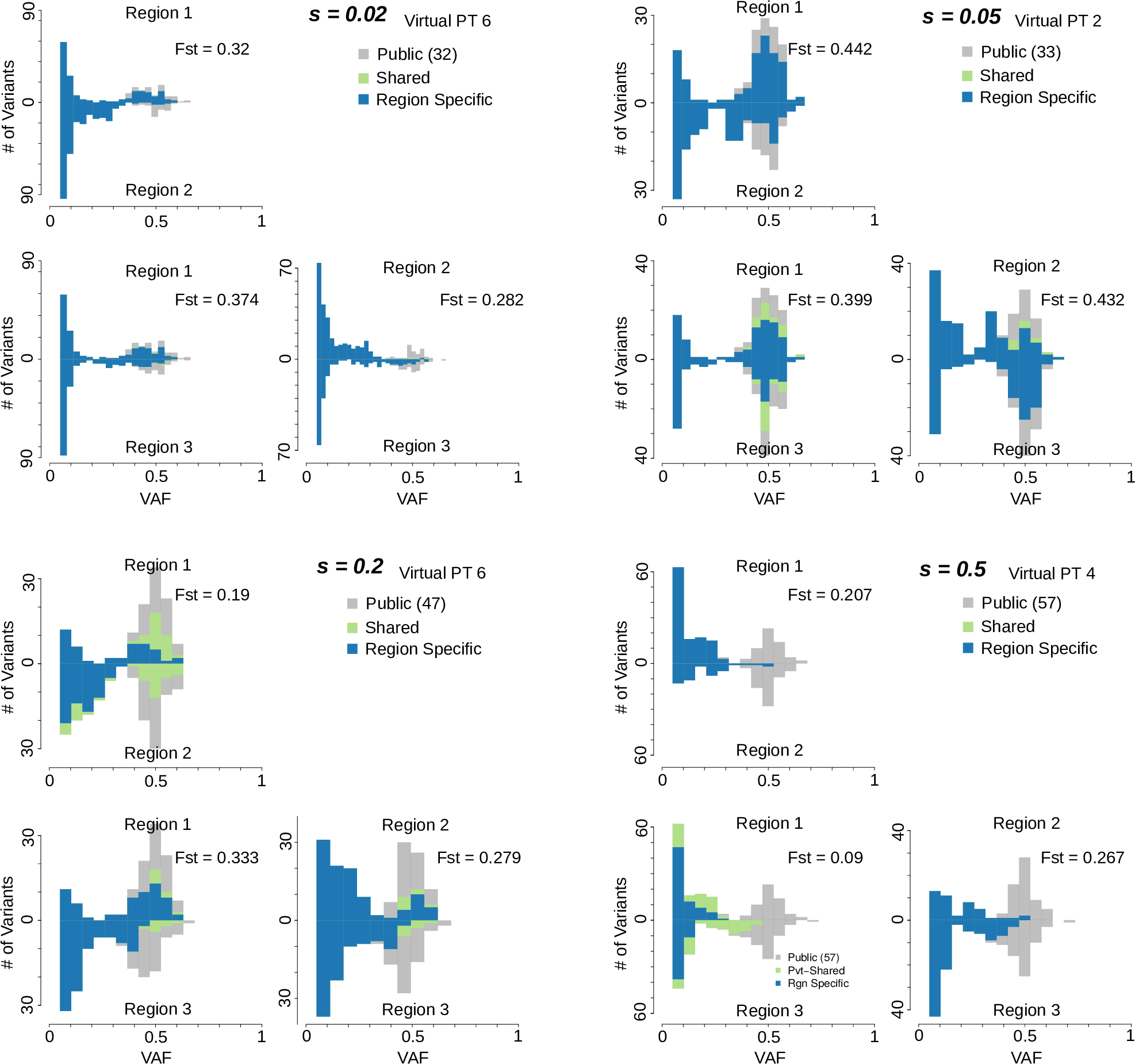

Supplement: S4 Fig — Representative pairwise VAF histograms derived from three spatially separated regions (labeled Region 1 to 3) within the same virtual primary tumor (PT) are shown for tumors simulated under different evolutionary modes. The pairwise histograms illustrate the number of variants detected at a given VAF for the two tumor regions shown above and below the x axis. The bin width was optimized for visualization purposes on the basis of the number of variants [72]. Variants were classified as public (gray), shared (green, subclonal variants shared in the two regions in comparison), or region specific (blue) on the basis of their presence in the virtual multi-region sequencing (5 regions in total) [18]. The number of public variants, as well as the Fixation index (Fst) are indicated (see Methods). Whereas the between-region genetic divergence increases when we raise the selection coefficient from 0.02 to 0.05, raising the selection coefficient further facilitates late subclonal sweeps, which can decrease the between-region genetic divergence. Note the increasing number of public variants and decreasing Fst on the lower panel (s = 0.2 and s = 0.5). (TIF) [file pcbi.1008838.s005.tif]

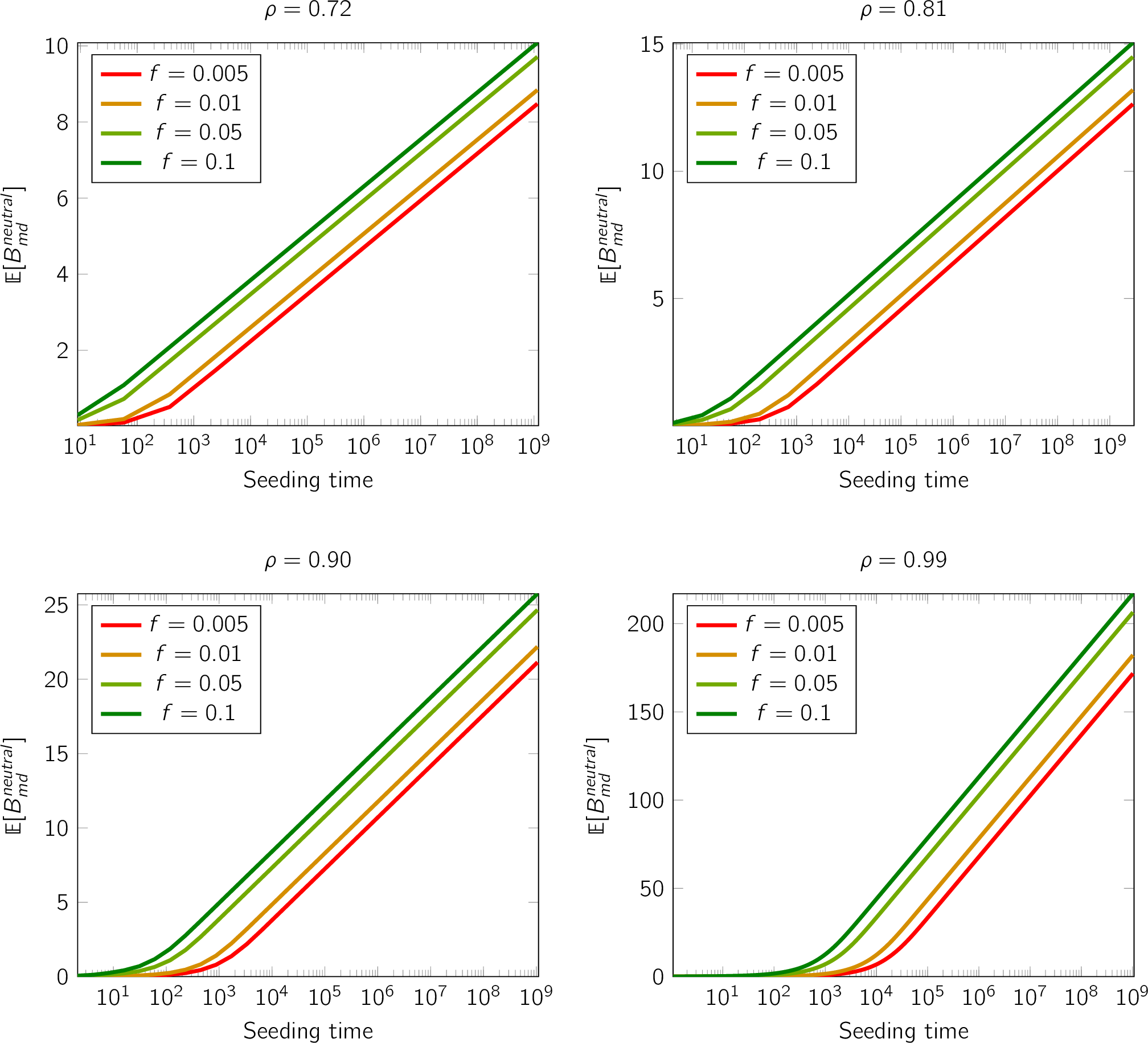

Supplement: S5 Fig — (TIF) [file pcbi.1008838.s006.tif]

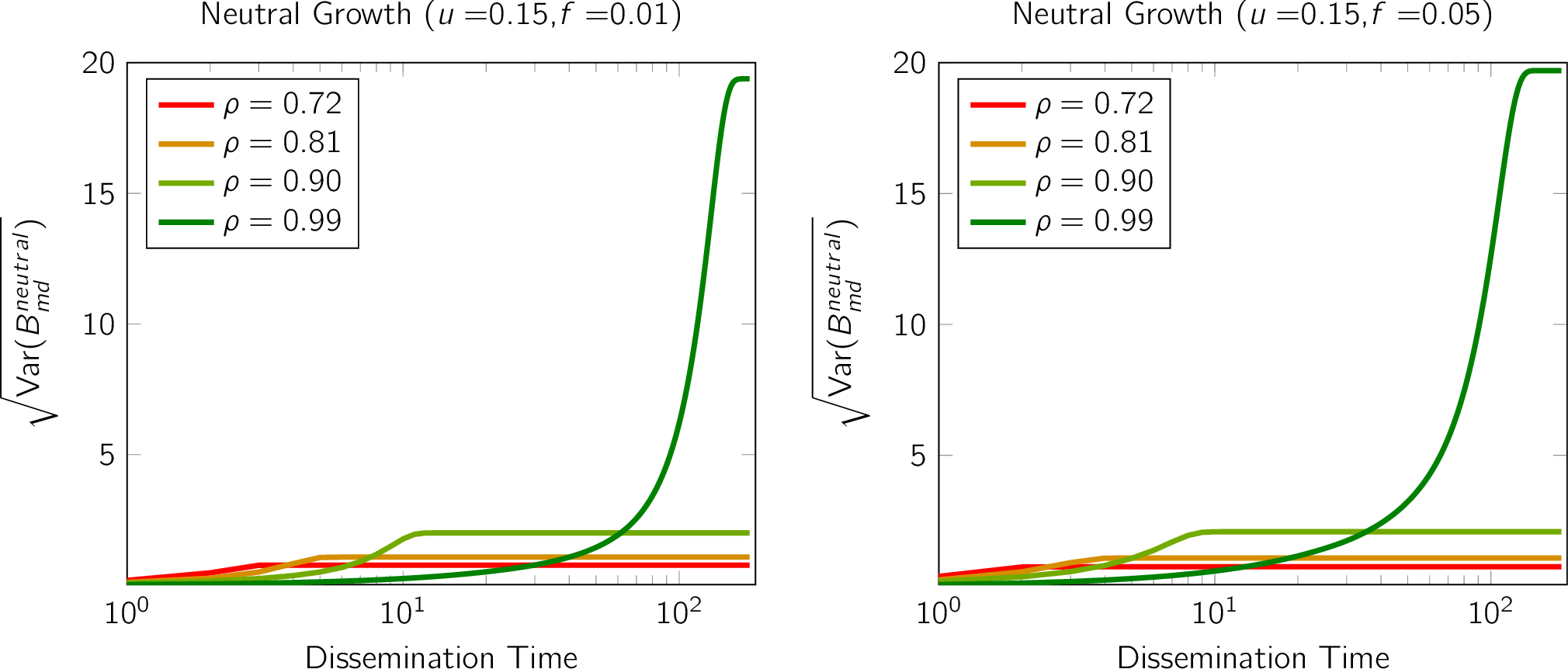

Supplement: S6 Fig — (TIF) [file pcbi.1008838.s007.tif]

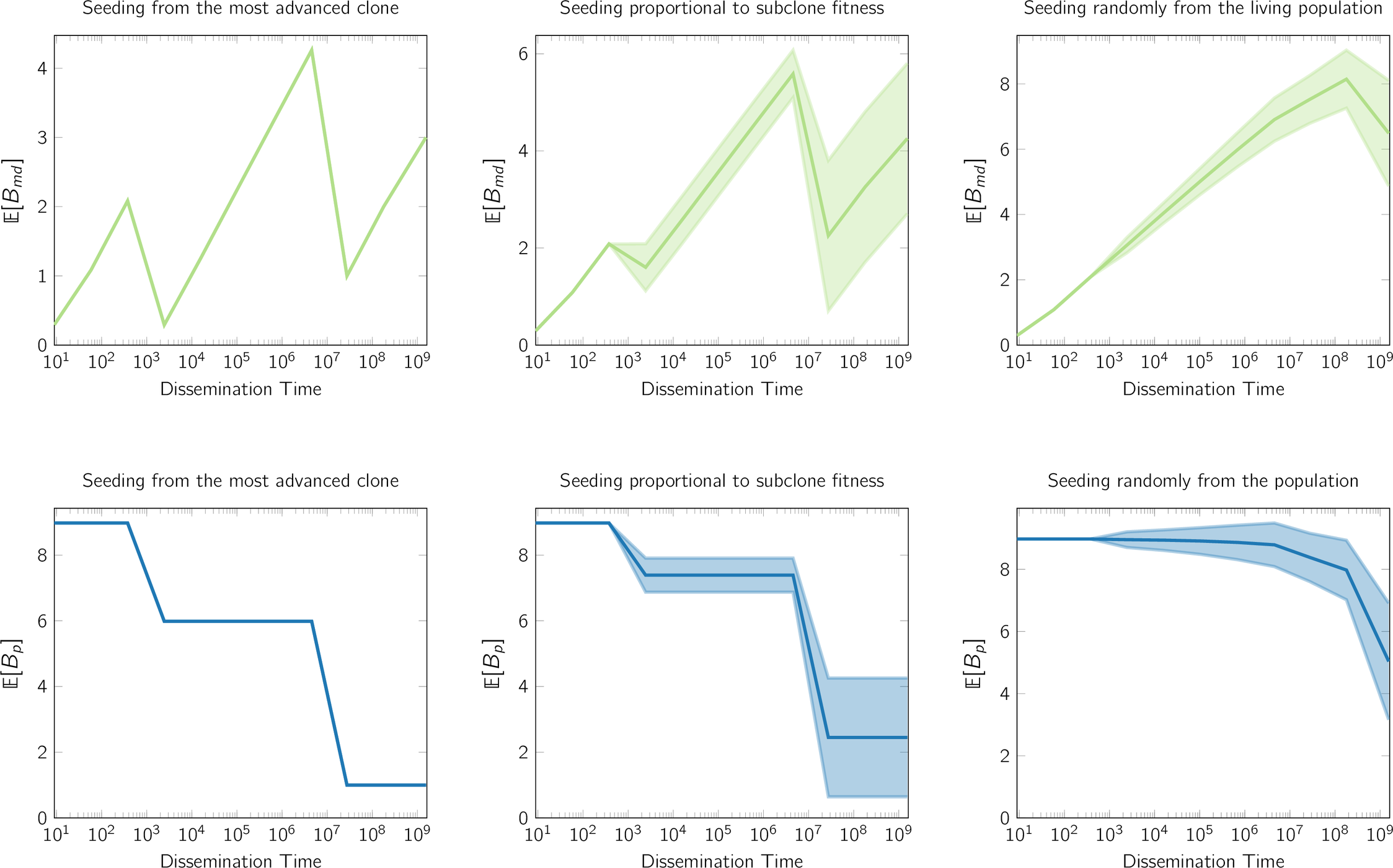

Supplement: S7 Fig — The growth parameters of the example are: ρ0 = 0.72; variant probability: u = 0.15; detectability frequency: f = 0.02; fitness of type 1: ρ1 = 0.64; fitness of type 2: ρ2 = 0.12. The population at detection time is 1.95e9; type-1 mutation appears in 41.18% of the population; type-2 mutation appears in 20.49% of the population. (TIF) [file pcbi.1008838.s008.tif]

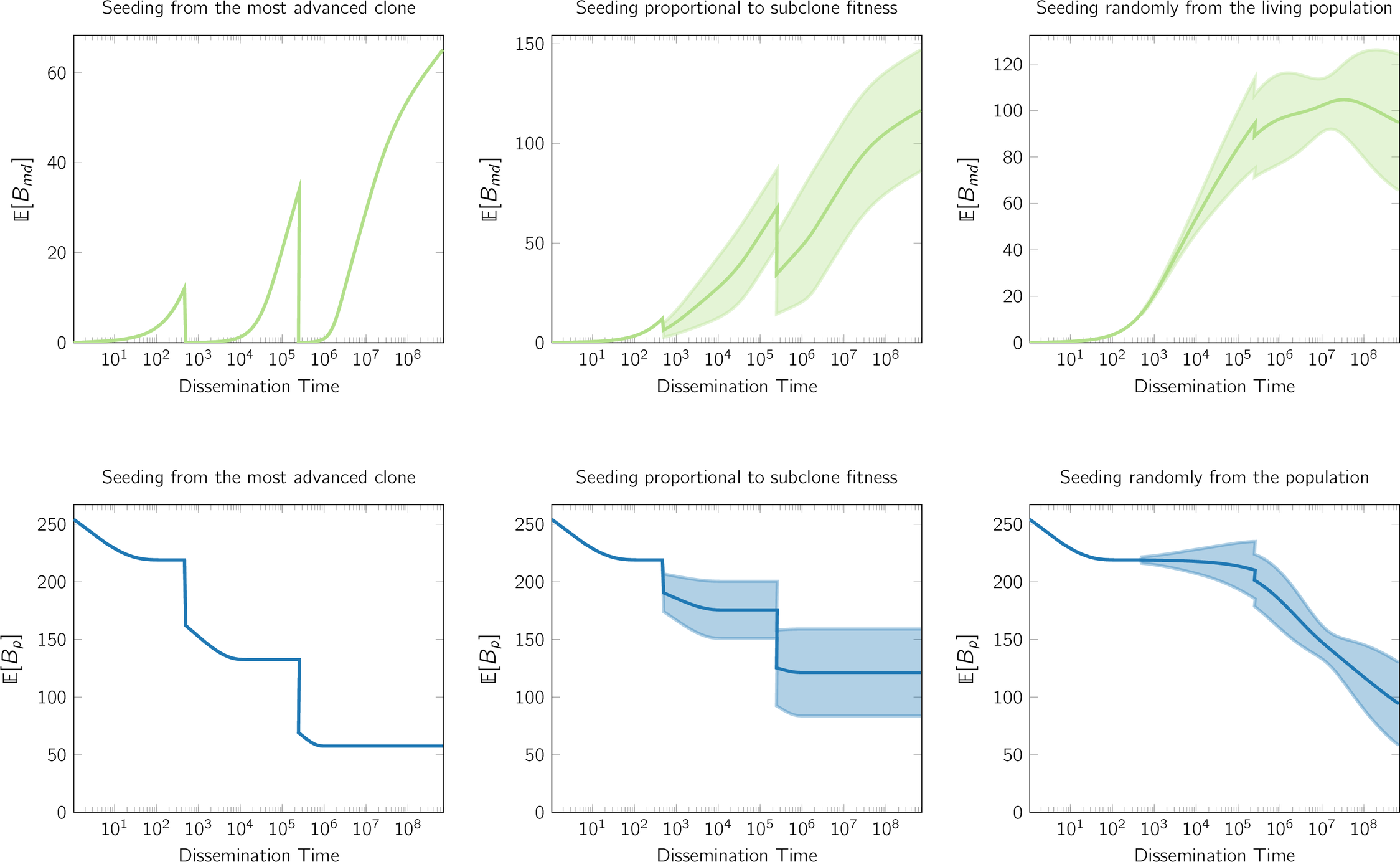

Supplement: S8 Fig — The growth parameters of the example are: ρ0 = 0.99; variant probability: u = 0.15; detectability frequency: f = 0.02; fitness of type 1: ρ1 = 0.984; fitness of type 2: ρ2 = 0.967. The population at detection time is 1.30e9; type-1 mutation appears in 91.84% of the population; type-2 mutation appears in 44.48% of the population. (TIF) [file pcbi.1008838.s009.tif]

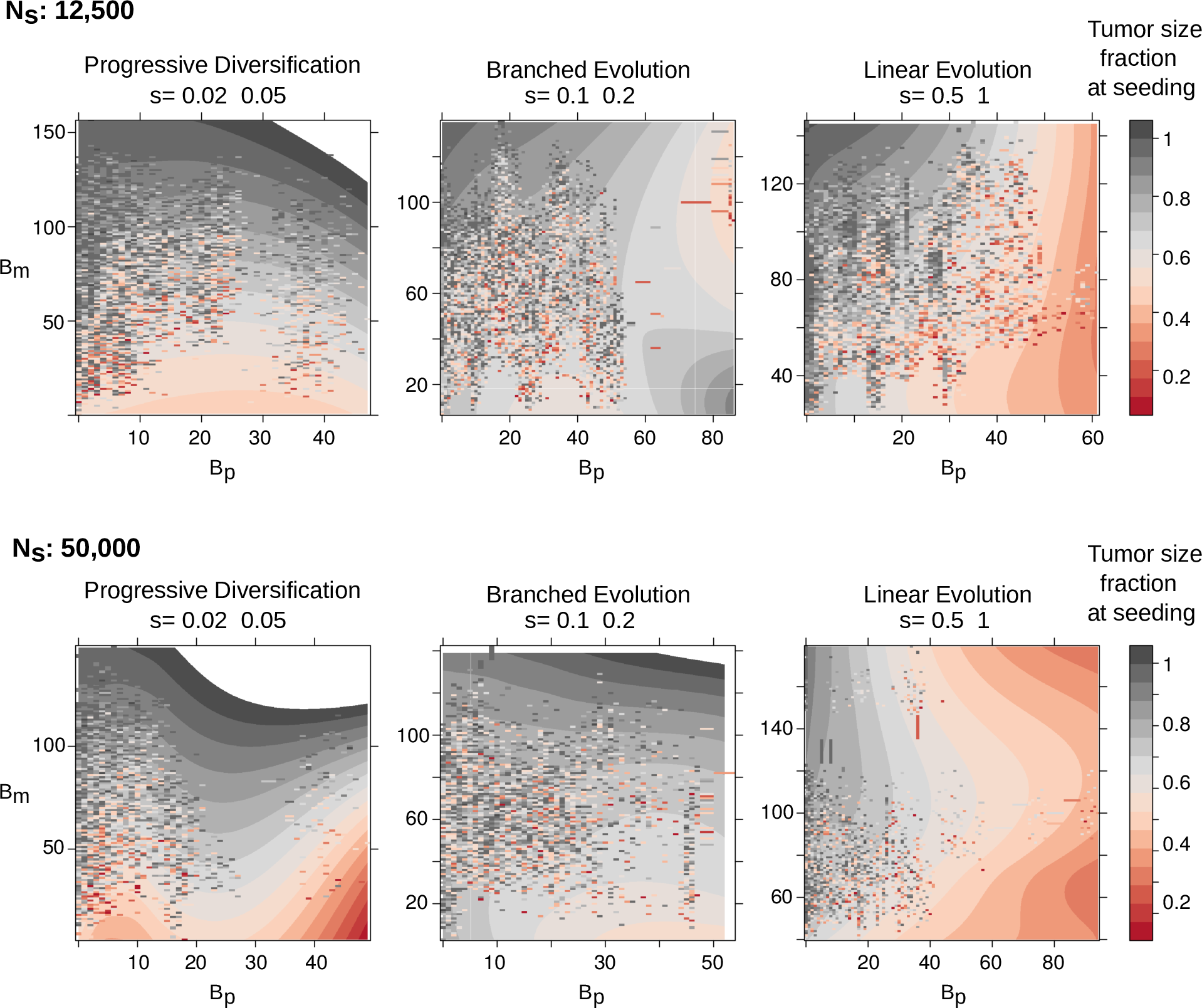

Supplement: S9 Fig — The upper panel: Ns = 12, 500, i.e., a half of the original setting used in the main manuscript; the lower panel: Ns = 50, 000, i.e., a double of the original setting. We group the various simulations into three representative kinetics of primary tumor growth: progressive diversification (s ≤ 0.05), branched evolution (s = {0.1, 0.2}) and linear evolution (s ≥ 0.5). For each kinetics, the actual seeding time is shown as color scales on top of the corresponding Bm and Bp values. A smoothed layer is added to show the general distribution of the seeding time. (TIF) [file pcbi.1008838.s010.tif]

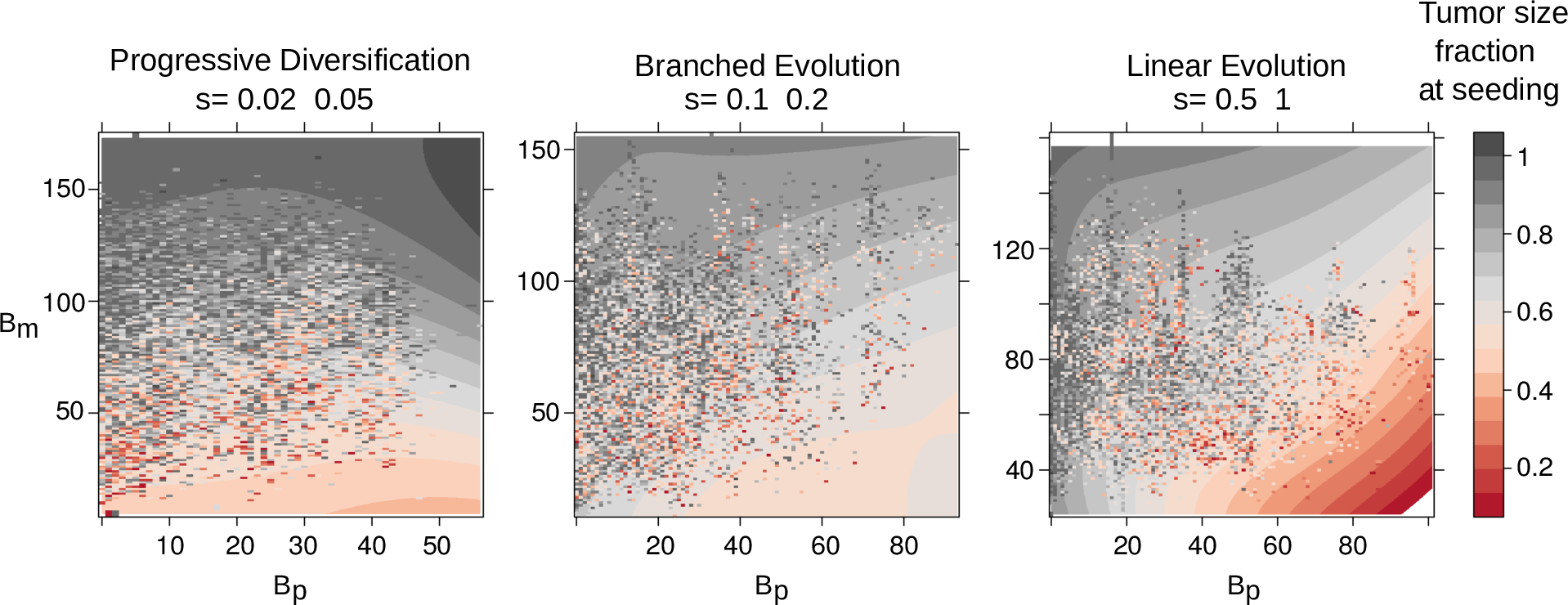

Supplement: S10 Fig — We group the various simulations into three representative kinetics of primary tumor growth: progressive diversification (s ≤ 0.05), branched evolution (s = {0.1, 0.2}) and linear evolution (s ≥ 0.5). For each kinetics, the actual seeding time is shown as color scales on top of the corresponding Bm and Bp values. A smoothed layer is added to show the general distribution of the seeding time. (TIF) [file pcbi.1008838.s011.tif]
