# Supplementary material for: Elements and evolutionary determinants of genomic divergence between paired primary and metastatic tumors
Source: PLoS Comput Biol. 2021 Mar 17;17(3):e1008838. doi: 10.1371/journal.pcbi.1008838 (PMC8007046; doi:10.1371/journal.pcbi.1008838)
Supplement: Attachment — Submitted filename: review.pdf [file pcbi.1008838.s012.pdf]

General comment

In their manuscript, "Elements and Evolutionary Determinants of Genomic Divergence Between Paired Primary and Metastatic Tumors" (PCOMPBIOL-D-20-01821) Ruping Sun and Athanasios Nikolakopoulos study how the growth mode of a primary tumor affects features of metastatic and primary tumor divergence. This paper is paving a theoretical groundwork for future disentanglement of relationship between metastases and their primary tumor. The importance of their work lies in showing that without knowing the primary tumor growth dynamics, one can not infer the time of dissemination of studied metastasis. Their insight will likely affect many studies that deal with relationship between metastatic and associated primary tumor. The theoretical methodology used appears sound and appropriate to draw the conclusions. However, even though we work in this specific field, we had a hard time understanding the model outline/parameters without a significant amount of work on our part to clarify. We therefore believe the paper would benefit from a significant restructuring of the introductory materials to make it more understandable.

Remarks (in no particular order)

1. The introduction of the manuscript reads nicely and authors present the central problem in a clear and understandable manner. The literature is well cited and is up to date with current trends in the field. However, we suggest authors to take a look at the following literature to get even broader overview of the field that models cancer evolution. Noble et al. 'Spatial structure governs the mode of tumour evolution'; https://www.biorxiv.org/content/10.1101/586735v2 or another similar paper from West et al.: 'Tissue structure accelerates evolution: premalignant sweeps precede neutral expansion': https://www.biorxiv.org/content/10.1101/542019v2. We too have done work on similar concepts – in particular thinking about the statistical metrics of the phylogenetic trees of tumors related to the underlying growth dynamics. Scott et al. Sys Bio 2019 https://doi.org/10.1093/sysbio/syz070. On this point, some metrics of the underlying trees from different simulations could be a useful adjunct to the analysis.

2. The authors haven't fully used the opportunity to introduce us to the underlying principles of their work and to teach us everything that is needed to fully understand the results. Figure 1 is minimal and doesn't really display what is going on in the paper. We suggest adding additional introductory figure that would include a cartoon of a cancer simulation in different growth stages and under different evolutionary regimes. Ideally, from this figure we would learn the rules of a spatial model: what does cell birth/death mechanism looks like, is there a inter-tumoral migration, how is spatial sampling conducted, why later variants are not detected in the sequencing etc. At the same time, simulation parameters and newly established M-P divergence measures can be introduced visually. Same cartoons of spatial simulation can also be used to better explain results, eg. figure 2b, top panel, since they are a product of underlying principles of the model.

   This is particularly important for colleagues who are new to the field. It will make much easier to visualize the dynamics of accumulation of mutations and difference in apparent number of mutations versus real number of mutation.

   If you do decide to leave current Figure 1 as a second figure or if you decide to merge it with new introductory figure, here are some comments on Fig1 in its current form. **Figure1**: This figure is crucial for understanding of entire concept so here are some suggestions on how to improve it. **Panel A):** Please specify what α and γ are since it is not clear from the figure nor from the caption.

   Panel B): We suggest to somehow mark the subclonal expansion of pre-seeding population so that it becomes clear why the ancestor of SC becomes detectable in third example.

   Panel C): you can add "dissemination" somewhere to better specify what "Late" and "Early" is. And also maybe mark "Bmd increases/drops" like in panel B. Maybe it would be good to replace "subclonal divergence" with "neutrally evolving tumour" and "subclonal convergence" with "tumour under selection." Or just append this info somewhere. These suggestions do increase the amount of information on the figure, which might be distracting, but we think it would make the subsequent figures easier to understand. I often think of the words of my collaborator and mentor, Steven Strogatz, when he told me that "each figure should

be considered an opportunity to teach the reader to better understand the current, and subsequent results."

3. We cannot find a link to the git repository of the modified *Comet* software used to produce the results. In our opinion it is of the utmost importance that the community should be able to repeat the calculations found in the paper. Therefore, putting your code to github or some other open repository is requested.

4. It seems that parameters N_s which is *tumor size increment to trigger seeding* and maximal tumour size N are taken arbitrarily. While we understand that the results in this paper are qualitative, and any one parameter change would neither validate or invalidate these results, it is important that we have confidence that the authors haven't found a rarity that exists only in a special slice of parameter space, but instead a somewhat general phenomenon. Authors should verify that different values of these parameters don't change their output much.

5. It is mentioned that there are others part of the metastatic cascade that are not considered. This is fine, but the authors should refer the reader to a systematic review. We have written one that could be used, but there are others. ours is: https://link.springer.com/chapter/10.1007/978-94-007-7835-1_9 here but it is by no means the only or the best/most up to date. Another more cartoon like one is here: https://www.nature.com/articles/nrc3287

6. α and γ are not really clear to me. Well, you explained nicely what they are, but I don't see why you introduced γ in the first place. Would it be a problem for the model if you would consider a mutation "substantially present", but not detected, if it is present already in one cell (γ being one step above 0)?

7. **Figure2**: Mark what s and ρ are since it is not stated in the caption. Fig 2B, bottom panel: append B_{md} to the x-axis label.

8. It would be good to again define every parameter in Materials and methods for readers who read this section before Results (possible in a table or 'quick reference box'.

9. the 'distance from center' metric used (often) seems to us to be a confounded choice. Without better understanding the model (which as we mentioned in the earlier comments) it is difficult to understand its utility – one could assume that the distance correlates with something more important (like, mutational burden?, divisional 'age'?) and that it is being used as a surrogate. However, since we are told there is death, this won't be a perfect concordance as there could be turnover in the bulk of the *in silico* tumor – but the reader is left to wonder these things. A better description of the model, or some sort of correlation/explanation of this metric would be helpful.

Conclusion

Overall, we found this paper really interesting and we see it as important contribution to the field of evolutionary oncology. As previously stated, improvement to the presentation and verification of used parameters would improve the manuscript greatly. We would be happy to see a revised version of the manuscript if the editors agree with our assessment.

Jacob G. Scott and Luka Opasic

P.S. We hope that the editors and authors stay safe and healthy during this trying time – there is light at the end of the tunnel!!
