# Supplementary material for: Elements and evolutionary determinants of genomic divergence between paired primary and metastatic tumors
Source: PLoS Comput Biol. 2021 Mar 17;17(3):e1008838. doi: 10.1371/journal.pcbi.1008838 (PMC8007046; doi:10.1371/journal.pcbi.1008838)
Supplement: Attachment — Submitted filename: Author_response_to_review_comments_MPD_RS_ANN_PLOSCB.pdf [file pcbi.1008838.s013.pdf]

Authors' Response to Reviews of

Elements and Evolutionary Determinants of Genomic Divergence Between Paired Primary and Metastatic Tumors

Ruping Sun and Athanasios N. Nikolakopoulos
PLOS Computational Biology, PCOMPBIOL-D-20-01821

RC: ***Reviewers' Comment***, AR: Authors' Response, ☐ Manuscript Text

General Comments

We appreciate the insightful, constructive critiques of our manuscript and were pleased to see that the reviewers acknowledge the importance of our work, noting that it paves a theoretical ground work in interpreting the genomic divergence between longitudinal tumor samples. After reading the comments, it becomes apparent to us that while there was significant enthusiasm for this work, aspects of the study design as well as the specific implications of our findings could have been made more evident. For example, it was not clear that computational modeling and mathematical modeling are two complementary ways to investigate the expected patterns of the M-P divergence. In this revision, we took the opportunity to take into account all the reviewer's comments, and improve the clarity of the aforementioned aspects. This is an important point, and we have now provided new introductory figures, added new results, revised figures that were confusing, and re-written texts to reflect all of these changes, as presented within.

We are also grateful for the editors' suggestions on improving the presentation of our manuscript in light of the reviewers' comments. Importantly, we now integrated the specific responses to their comments into our manuscript under the appropriate context, facilitating the overall flow of the central message of our work. We believe that the revised manuscript is substantially improved overall, and indeed, more accessible to people outside our area of specialisation.

Note: Line numbers refer to the revised manuscript version with changes being highlighted.

1. Response to Reviewer #1

RC: ***The authors investigated how the observed genetic divergence between paired primary and metastatic tumors is regulated. I believe the purpose of this paper is scientifically and medically meaningful to understand the genetic relationship between primary and metastatic tumors from paired genomic data that infers the time of seeding, which might lead to a proposal of the best treatment option at diagnosis in the future. They performed mathematical analysis and computational simulations separately and revealed that dissemination from an advanced detectable subclone of the primary tumor can lead to an abrupt drop in the expected measurable M-P divergence, thereby breaking the previously assumed monotonic relation between seeding time and M-P divergence. I am not convinced with their major finding stated above because they showed two kinds of results derived from mathematical analysis and computational simulations separately and then they insisted these are consistent without comparing them in the same panel. I would like to see clear correspondence between mathematical predictions and computational simulations with the same parameter values in the same panel. After that, the analytical insights become convincing to support the conclusion.***

AR: We thank the reviewer for the recognition of the motivation and the clinical implication of our work. While our mathematical modeling (without spatial constraints) and the computational simulation (with spatial constraints) explore complementary model space for unravelling the general fashion on how evolutionary forces regulate M-P divergence, we found that the nature of our study design can be made more evident and the two approaches can be connected tighter. It should be noted, however, that it is the growth dynamics, not the model-dependent parameter values, that would condition the relationship between the seeding time and M-P divergence. As such, we believe that aligning the patterns of growth rather than the specific parameter values used in two distinct model settings is more relevant to our findings. We further emphasized this point in the revised manuscript to avoid potential misunderstandings. In this revision, we introduced a new figure highlighting our study design (Fig 1). Besides, we now added new figure panels to the original Fig 4 (now Fig 5A and 5C), to facilitate the reading of the results of spatial simulation by aligning the mathematical insights which illuminates the growth dynamics and math predictions. Finally, we tested in the computational simulations the key prediction of our mathematical analysis, i.e., the "drop" of M-P divergence due to the subclonal expansion (Fig 5C). These new results suggest that both our approaches point to the same conclusion (line 278-282).

RC: ***Moreover, their conclusion that a non-monotonic relationship appears under branched and linear evolution does not have a big impact since it is just a denial, rather I expect the authors propose a rule of the genetic divergence between primary and metastatic tumor under such evolution.***

AR: Our framework does indicate the general association between the drops of B_{md} and subclonal expansion (to detectable sizes) under selective models, as further supported by our new analyses (Fig 5C). Respectfully, we do not view the non-monotonicity result as a “denial". Our work, rather, aims to highlight the circumstances (as well as the underlying growth assumptions) under which non-monotonicity between M-P divergence and metastatic seeding time is to be expected (as emphasized at line 60-72 in the revised manuscript). Our work does not exclude monotonic dependency, which is expected in the neutral growth setting as well as under "fitness-oblivious" seeding mechanisms (see our discussion in 4.1.3). We believe that our findings illuminate the intrinsic complexities of the problem under study and call for a careful reading of the M-P divergence as the metastatic seeding patterns in practice. Due to these reasons, we now emphasized the finding that subclonal expansion leads to reduction of divergence in the first paragraph of Discussion, along with several other rules uncovered by our constructive framework (line 311-316). We wrote as:

> (1) Depth of most recent detectable variant characterizes the M-P divergence; (2) Decay in the probability of a variant being detectable by sequencing determines the expected M-P divergence; (3) Dissemination from late detectable subclone leads to an abrupt drop in M-P divergence and (4) Spatial model verifies that the growth mode governs the dependence of M-P divergence on seeding time.

By illuminating the fundamental evolutionary determinants of M-P divergence, our findings can be viewed as the first step toward a more reliable connection between the genomic heterogeneity measures and underlying tumor evolutionary processes. As the interpretation of such genomic heterogeneity measure could affect clinical decision making, we hope that our findings will facilitate future research to build more sophisticated quantitative framework to fully decompose the complexity of cancer progression.

2. Response to Reviewer #2

2.1. General comment

RC: ***In their manuscript, “Elements and Evolutionary Determinants of Genomic Divergence Between Paired Primary and Metastatic Tumors” (PCOMPBIOL-D-20-01821). Ruping Sun and Athanasios Nikolakopoulos study how the growth mode of a primary tumor affects features of metastatic and primary tumor divergence. This paper is paving a theoretical groundwork for future disentanglement of relationship between metastases and their primary tumor. The importance of their work lies in showing that without knowing***

the primary tumor growth dynamics, one can not infer the time of dissemination of studied metastasis. Their insight will likely affect many studies that deal with relationship between metastatic and associated primary tumor. The theoretical methodology used appears sound and appropriate to draw the conclusions. However, even though we work in this specific field, we had a hard time understanding the model outline/parameters without a significant amount of work on our part to clarify. We therefore believe the paper would benefit from a significant restructuring of the introductory materials to make it more understandable.

AR: First we would like to thank the reviewer for their recognition of the importance and implications of our findings. The comments are very helpful, we now integrated the specific responses to these comments into our manuscript under the appropriate context, facilitating the overall flow of the message (see below).

2.2. Remarks (in no particular order)

RC: ***The introduction of the manuscript reads nicely and authors present the central problem in a clear and understandable manner. The literature is well cited and is up to date with current trends in the field. However, we suggest authors to take a look at the following literature to get even broader overview of the field that models cancer evolution. Noble et al. 'Spatial structure governs the mode of tumour evolution'; `https://www.biorxiv.org/content/10.1101/586735v2` or another similar paper from West et al.: 'Tissue structure accelerates evolution: premalignant sweeps precede neutral expansion': `https://www.biorxiv.org/content/10.1101/542019v2`. We too have done work on similar concepts – in particular thinking about the statistical metrics of the phylogenetic trees of tumors related to the underlying growth dynamics. Scott et. al. Sys Bio 2019 `https://doi.org/10.1093/sysbio/syz070`. On this point, some metrics of the underlying trees from different simulations could be a useful adjunct to the analysis.***

AR: We thank the reviewer for their thorough review of the introduction section. Indeed, Noble's insightful pre-print had already been mentioned in the original paper. Besides Nobel's work, we now referred to West et al.'s finding of the impact of spatial constraints on the effectiveness of selection in the revised manuscript (see line 34). The theoretical piece in Sys. Bio. (2019) is also a nice reference for us to introduce our motivation better. Hence, we now have referred to this work in the revised manuscript under the appropriate context (see line 67-72).

RC: ***The authors haven't fully used the opportunity to introduce us to the underlying principles of their work and to teach us everything that is needed to fully understand the results. Figure 1 is minimal and doesn't really display what is going on in the paper. We***

suggest adding additional introductory figure that would include a cartoon of a cancer simulation in different growth stages and under different evolutionary regimes. Ideally, from this figure we would learn the rules of a spatial model: what does cell birth/death mechanism looks like, is there a inter-tumoral migration, how is spatial sampling conducted, why later variants are not detected in the sequencing etc. At the same time, simulation parameters and newly established M-P divergence measures can be introduced visually. Same cartoons of spatial simulation can also be used to better explain results, eg. figure 2b, top panel, since they are a product of underlying principles of the model.
This is particularly important for colleagues who are new to the field. It will make much easier to visualize the dynamics of accumulation of mutations and difference in apparent number of mutations versus real number of mutation.

AR: We really appreciate the suggestions on improving the introduction of our approaches. To this end, we made a new Fig 1 outlining the study design. In this figure, we presented example visual illustrations showing tumor evolution and sampling process; we highlighted the main question by introducing the M-P divergence and the potential evolutionary determinants. Moreover, we visually presented the two complementary approaches for tackling this question, i.e., the mathematical modeling and spatial computational simulation. Some detailed aspects are difficult to visualize, (such as the rules of spatial constraints in the computational modeling) hence we mention them in the figure legends, and refer the readers to the Method section.

RC: ***If you do decide to leave current Figure 1 as a second figure or if you decide to merge it with new introductory figure, here are some comments on Fig1 in its current form. Figure1: This figure is crucial for understanding of entire concept so here are some suggestions on how to improve it. Panel A): Please specify what α and γ are since it is not clear from the figure nor from the caption.***

AR: Done. See the updated figure (now Fig 2).

RC: ***Panel B): We suggest to somehow mark the subclonal expansion of pre-seeding population so that it becomes clear why the ancestor of SC becomes detectable in the third example.***

AR: Done. See the updated figure (now Fig 2).

RC: ***Panel C): you can add "dissemination" somewhere to better specify what "Late" and "Early" is. And also maybe mark "Bmd increases/drops" like in panel B. Maybe it would be good to replace "subclonal divergence" with "neutrally evolving tumour" and "subclonal convergence" with "tumour under selection." Or just append this info somewhere. These suggestions do increase the amount of information on the figure, which might be***

distracting, but we think it would make the subsequent figures easier to understand. I often think of the words of my collaborator and mentor, Steven Strogatz, when he told me that "each figure should be considered an opportunity to teach the reader to better understand the current, and subsequent results."

AR: We thank the referee for their careful attention to the details and giving constructive suggestions about improving the message flow of the figures. We now made changes to this figure accordingly (see Fig 2).

RC: ***We cannot find a link to the git repository of the modified Comet software used to produce the results. In our opinion it is of the utmost importance that the community should be able to repeat the calculations found in the paper. Therefore, putting your code to github or some other open repository is requested.***

AR: We now have made the repository public.
(`https://github.com/SunPathLab/Comet`)

RC: ***It seems that parameters Ns which is tumor size increment to trigger seeding and maximal tumour size N are taken arbitrarily. While we understand that the results in this paper are qualitative, and any one parameter change would neither validate or invalidate these results, it is important that we have confidence that the authors haven't found a rarity that exists only in a special slice of parameter space, but instead a somewhat general phenomenon. Authors should verify that different values of these parameters don't change their output much.***

AR: We now ran simulations with two additional N_s, 12,500 and 50,000, i.e., a half and a double of the original setting (25,000), respectively. The mode-dependent pattern of M-P divergence is reproducible in these new simulation runs (S9 Fig, line 302, 669).

RC: ***It is mentioned that there are others part of the metastatic cascade that are not considered. This is fine, but the authors should refer the reader to a systematic review. We have written one that could be used, but there are others. ours is: `https://link.springer.com/chapter/10.1007/978-94-007-7835-1_9` here but it is by no means the only or the best/most up to date. Another more cartoon like one is here: `https://www.nature.com/articles/nrc3287`***

AR: In this revision, we now introduce the literature regarding mathematical models of those other parts of metastatic cascade, including what have been suggested by the reviewer (line 86-87 and 418-421). We thank the reviewers for this suggestion.

RC: α ***and*** γ ***are not really clear to me. Well, you explained nicely what they are, but I don't see why you introduced*** γ ***in the first place. Would it be a problem for the model if***

you would consider a mutation "substantially present", but not detected, if it is present already in one cell (γ being one step above 0)?

AR: γ is greater than α. The threshold γ is oriented from the perspective of quantifying the genomic divergence given the noise of bulk sequencing data. For a variant to be considered as condition-specific (i.e., primary- or metastatic-specific), one would avoid overestimating the divergence (e.g., control for false positive rates) by requiring that (1) the variant allele frequency (VAF) to be higher than a threshold γ suggesting the substantial presence in a condition; and (2) the VAF in the other condition is lower than a threshold α suggesting the absence. We now emphasized this consideration in the revised manuscript (see line 97-103).

RC: ***Figure2: Mark what s and ρ are since it is not stated in the caption. Fig 2B, bottom panel: append Bmd to the x-axis label.***

AR: Done (see Fig 3). As the new Fig 1 introduces these parameters first, these symbols should be easier to digest. We appreciate the considerate comments of the reviewer.

RC: ***It would be good to again define every parameter in Materials and methods for readers who read this section before Results (possible in a table or 'quick reference box'.***

AR: Done. See updated Table 1.

RC: ***the 'distance from center' metric used (often) seems to us to be a confounded choice. Without better understanding the model (which as we mentioned in the earlier comments) it is difficult to understand its utility – one could assume that the distance correlates with something more important (like, mutational burden?, divisional 'age'?) and that it is being used as a surrogate. However, since we are told there is death, this won't be a perfect concordance as there could be turnover in the bulk of the in silico tumor – but the reader is left to wonder these things. A better description of the model, or some sort of correlation/explanation of this metric would be helpful.***

AR: We appreciate this insightful comment. The distance to center is chosen as one of the two axes for "separating" the seeding cell lineages apart for visualizing the "tree in space" of tumor growth, where seeding cell lineage and detectable (sampled) cells are marked. It should be noted that in our spatial model, virtual tumor largely follows a peripheral growth pattern. As the primary tumor expands, the cells will inevitably reach outer spaces. It appears to be a natural choice for making a two dimensional visual representation of the tumor growth with numerous lineages. This metric is indeed correlated with mutation burden (see S3 Fig). We now also commented on this in the main manuscript (legend of Fig 3B and Fig 5B).

2.3. Conclusion

RC: ***Overall, we found this paper really interesting and we see it as important contribution to the field of evolutionary oncology. As previously stated, improvement to the presentation and verification of used parameters would improve the manuscript greatly. We would be happy to see a revised version of the manuscript if the editors agree with our assessment.***

AR: We thank the reviewer for their thorough assessment of our work and for giving highly complimentary remarks. We are glad that the value of our work is recognised by experts who work in the same field. We also found that the critiques were constructive, and have served to substantially improve the clarity of our paper and increase the potential impact of our study.

3. Response to Reviewer #3

3.1. Major Concerns

RC: ***Overall this is a nicely written paper that needs some minor modifications before being acceptable for publication. First off, I'm surprised that the authors do not expound more about multi-region sequencing in the last paragraph of the introduction. Surely the use of this approach that is common in the literature of studying evolutionary dynamics in primary tumors is worth mentioning its benefits and limitations as well. Especially since this is discussed briefly in the discussion (lines 305-307 and again in lines 318-320). I argue that multi-region sequencing has begun to ensure that spatial sampling locations are preserved, albeit with poor resolution, but exists, nonetheless.***

AR: We thank the referee for his recognition of the quality of our paper and for commenting on the availability of longitudinal genomic sequencing data with spatial sampling. In this revision, we not only expanded a bit more to mention the increased focus on collecting such datasets in the field of cancer genomics (and citing relevant papers where spatial information is indeed reported), but also use the opportunity to highlight the challenges in interpreting heterogeneity measures from such data, in order to connect better with our motivation behind laying the theoretical groundwork for understanding the evolutionary determinants of the observed divergence (see line 60-72, Fig 1). We also added the "poor resolution" in commenting on the current status of tracking the spatial location of bulk sampling in the Discussion (line 354).

RC: ***Line 329-333 the authors use poor resolution sample heterogeneity estimates as justification for their lack of comparisons between real data and computational models. While this is certainly true. The authors should include a sentence to propose how this could***

be resolved in future studies to begin to think about how data can be used to validate the authors primary findings and justifications.

AR: We thank the reviewer for the understanding of challenges associated with data itself. As we have stated in the manuscript, a quantitative assessment of the effect of suboptimal detection (sampling size, purity differences and ploidy changes, etc), on the observed genomic heterogeneity is necessary, before comparing the real data with computational models (line 366-368). We are hopeful that the emerging technologies could provide better opportunities to validate our findings in real patient data. The combination between longitudinal bulk sampling and single-cell sequencing would provide the ultimate resolution to distinguish the tumor growth dynamics and track the cell genealogy, hence would enable one to test hypotheses generated by our model (line 368-372). Lastly, as our results suggest that the mode of tumor growth conditions the interpretation of M-P divergence, investigating the detailed growth dynamics in patient tumors would increase the utility of genomic heterogeneity measures (line 423-426).

RC: ***The authors state, based on citation 30, that the majority of distant mets are monoclonal. The citation of a single article is insufficient given several other studies that have shown different cancer types that result in various mets showing evidence for polyclonal seeding (e.g. Hirotsu 2020, Hu 2020). At minimum this should be discussed in greater detail. However, I feel it's necessary to have polyclonal seedings combined with the monoclonal seedings the authors describe. If the "analytical thinking is extendable" (Line 364 to 366), this should be done to provide greater insights into our understandings of metastasis.***

AR: We agree with the reviewer that polyclonal seeding is, indeed, an important direction; highly relevant for the field. However, a complete and thorough treatment of polyclonal seeding would require specialized modeling considerations that go beyond the scope of our current study. We believe that improving the understanding of monoclonal seeding—which is the basic goal of this work—is a crucial prerequisite that can pave the path for future approaches that can be generalized to the polyclonal seeding setting. In the revised version of our manuscript, we now outline potential ways to extend our model to study polyclonal seeding (see line 402-421), however a detailed study of such approaches is purposefully left as future work. Our related discussion in the revised manuscript reads:

> Poly-clonal seeding (or multi-cell seeding, under the strict definition of a clone) involves the following two scenarios: monophyletic (genetic homogeneity among the seeding cells) and polyphyletic (genetic heterogeneity instead) [1]. Our framework can be applied directly to monophyletic seeding (note that in terms of the resulting

M-P divergence, seeding from a single cell is genomically equivalent to the colonization by a group of cells in the monophyletic lineage). In contrast to monophyletic dissemination, polyphyletic seeding would lead to a reduction in M-P divergence as more genetic diversity in the primary is transmitted to the metastasis (e.g., suggested by subclonal sharing between primary and metastasis sequencing data [2], assuming that purity and local copy number differences are taken into account). Nevertheless, the tumor growth mode and seeding tempo are still expected to regulate the observed M-P divergence. For example, seeding simultaneously as a cluster, or consecutively could lead to distinct patterns in M-P divergence [3]. Our framework can be used to study the divergence between the primary and each seeding subclone characterised from sequencing data [4] but more specialized considerations are required to fully model the polyphyletic seeding. Moreover, here we do not model the circulatory phase of metastasis, a bottleneck that could confound the M-P divergence further. Unified mathematical models have been proposed to understand the full picture of this phase [5–7], which would aid the decomposition of the complexity of metastases.

RC: ***Figure 5A needs some adjustments to be interpretable. First and foremost move the selection coefficient outside of the plotted area or put a distinctive box around this. The points appear as part of the plot. The points in this legend should be aligned by the center of the circle as opposed to the bottom. For six discrete groupings of variance a more divergent colormap would aid in visualization. The intervals themselves within this plot are odd; why are they different? Jumping from 0.1 intervals to 0.05 intervals and back to 0.1 intervals?***

AR: We now updated this figure (see Fig 6A). We appreciate the reviewer's suggestions on improving the clarity of this figure. The color binning now is based on 10-quantile of all the values. One can see that under strong selective modes, the variance of B_m being explained by seeding time is less than 10 percent.

RC: ***Section 4.2.4. There seems (to be) a slight disconnect with the authors use of virtual sequencing versus the Hudson Fst estimate and the multi-region sampling above in 4.2.3. This isn't virtual sequencing in a way that can relate back to any real world data with technical error, sequencing depth, and attempts to being able to relate any of the metrics/calculations back to real data. This section would be better described only by 'Tumor genealogy analysis.' Virtual sequencing would be more akin to methods proposed by Williams et al. 2018 and several other papers. The descriptions in 4.2.3 where assumed negative binomial distribution with depth being accounted for are present here. What differs in these two sections in their samplings and assumptions for calculating VAFs?***

AR: We now updated the title of section 4.2.4 as Analysis of virtual genomic divergence and tumor genealogy. F_{st} calculation is based on the variant allele frequencies obtained from Section 4.2.3. In other words, the virtual sampling provides the input variant allele frequencies for F_{st} calculation. As this section describes the methods for calculating the virtual genomic divergence (between multi-region samples, and between paired virtual primary and metastatic tumors), we believe that the new section title should serve to connect the previous section better. We thank the reviewer for the suggestion on improving the clarity of our manuscript.

3.2. Minor Comments

RC: ***Remove the comma in the sentence starting on line 40 (A recently published, deme…).***

AR: Done (see line 40).

RC: ***Add a comma after however in line 298.***

AR: Done (see line 332).

References

[1] Birkbak NJ, McGranahan N. Cancer Genome Evolutionary Trajectories in Metastasis. Cancer Cell. 2020;37(1):8–19. doi:10.1016/j.ccell.2019.12.004.

[2] Hirotsu Y, Hada M, Amemiya K, Oyama T, Mochizuki H, Omata M. Multi-regional sequencing reveals clonal and polyclonal seeding from primary tumor to metastases in advanced gastric cancer. Journal of Gastroenterology. 2020;55(5):553–564. doi:10.1007/s00535-019-01659-6.

[3] Heyde A, Reiter JG, Naxerova K, Nowak MA. Consecutive seeding and transfer of genetic diversity in metastasis. Proceedings of the National Academy of Sciences of the United States of America. 2019;116(28):14129–14137. doi:10.1073/pnas.1819408116.

[4] Caravagna G, Heide T, Williams MJ, Zapata L, Nichol D, Chkhaidze K, et al. Subclonal reconstruction of tumors by using machine learning and population genetics. Nature Genetics. 2020;52(9):898–907. doi:10.1038/s41588-020-0675-5.

[5] Szczurek E, Krüger T, Klink B, Beerenwinkel N. A mathematical model of the metastatic bottleneck predicts patient outcome and response to cancer treatment. PLOS Computational Biology. 2020;16(10):e1008056. doi:10.1371/journal.pcbi.1008056.

[6] Scott J, Kuhn P, Anderson ARA. Unifying metastasis-integrating intravasation, circulation and end-organ colonization; 2012. Available from: http://labpages.moffitt.org/andersona/.

[7] Scott JG, Gerlee P, Basanta D, Fletcher AG, Maini PK, Anderson ARA. Mathematical modeling of the metastatic process. In: Experimental Metastasis: Modeling and Analysis. Springer Netherlands; 2013. p. 189–208. Available from: https://link.springer.com/chapter/10.1007/978-94-007-7835-1{_}9.
